# Estrous cycle-dependent changes of Fas expression in the bovine corpus luteum: influence of keratin 8/18 intermediate filaments and cytokines

**DOI:** 10.1186/1477-7827-10-90

**Published:** 2012-10-31

**Authors:** Alice Duncan, Jennifer Forcina, Alyssa Birt, David Townson

**Affiliations:** 1 Department of Molecular, Cellular and Biomedical Sciences, University of New Hampshire, Durham, NH, USA; 2 Department of Dairy and Animal Science, The Pennsylvania State University, University Park, PA, USA

**Keywords:** Apoptosis, Corpus Luteum, Cytokines, Cytoskeleton, Fas, Ovary

## Abstract

**Background:**

Fas expression and Fas-induced apoptosis are mechanisms attributed to the selective destruction of cells of the corpus luteum (CL) during luteal regression. In certain cell-types, sensitivity to these death-inducing mechanisms is due to the loss or cleavage of keratin-containing intermediate filaments. Specifically, keratin 8/18 (K8/K18) filaments are hypothesized to influence cell death in part by regulating Fas expression at the cell surface.

**Methods:**

Here, Fas expression on bovine luteal cells was quantified by flow cytometry during the early (Day 5, postovulation) and late stages (Days 16–18, postovulation) of CL function, and the relationship between Fas expression, K8/K18 filament expression and cytokine-induced cell death *in vitro* was evaluated.

**Results:**

Both total and cell surface expression of Fas on luteal cells was greater for early versus late stage bovine CL (89% vs. 44% of cells for total Fas; 65% vs.18% of cells for cell surface Fas; respectively, P<0.05, n=6-9 CL/stage). A similar increase in the steady-state concentration of mRNA for Fas, as detected by quantitative real-time polymerase chain reaction, however, was not observed. Transient disruption of K8/K18 filaments in the luteal cells with acrylamide (5 mM), however, had no effect on the surface expression of Fas (P>0.05, n=4 CL/stage), despite evidence these conditions increased Fas expression on HepG2 cells (P<0.05, n= 3 expts). Exposure of the luteal cells to cytokines induced cell death (P<0.05) as expected, but there was no effect of K8/K18 filament disruption by acrylamide (P>0.05) or stage of CL (P>0.05, n= 4 CL/stage) on this outcome.

**Conclusion:**

In conclusion, we rejected our null hypothesis that the cell surface expression of Fas does not differ between luteal cells of early and late stage CL. The results also did not support the idea that K8/K18 filaments influence the expression of Fas on the surface of bovine luteal cells. Potential downstream effects of these filaments on death signaling, however, remain a possibility. Importantly, the elevated expression of Fas observed on cells of early stage bovine CL compared to late stage bovine CL raises a provocative question concerning the physiological role(s) of Fas in the corpus luteum, particularly during early luteal development.

## Background

The receptor molecule CD95 (Apo-1) or Fas, is considered an integral component of immune-response mechanisms within the corpus luteum (CL) which potentially influence luteal function. It is a member of the TNF receptor superfamily [1] and is thought of as the prototypical death receptor because when bound by Fas ligand (FasL), cells undergo apoptosis [2]. The binding of FasL to Fas triggers trimerization of Fas receptor on the cell surface. This complex then leads to the activation of Fas associated death domain and pro-caspase-8 proteins. The cleavage of pro-caspase-8 signals the caspase cascade, which then leads to the activation of pro-caspase-3 and apoptosis [3,4]. Indeed, in the cow, expression of Fas mRNA within the CL occurs throughout the luteal phase [5], and exposure of luteal cells to FasL, induces apoptosis [5,6]. Recently, Kliem and coworkers determined Fas and FasL mRNA increase in bovine CL within 30 min to 2 h of injecting cows with a luteolytic dose of prostaglandin F2-alpha [7], further supporting the death-inducing role of Fas and FasL in the CL. These observations collectively suggest Fas-induced mechanisms within the bovine CL constitute a plausible pathway for the cell-specific death observed during luteal regression.

The attractiveness of the Fas-induced death pathway in luteal regression is that it is relatively conserved among species and it provides for the selective elimination of cells (i.e., via apoptosis) without invoking an inflammatory response. Indeed, regression of the CL is characterized by cells undergoing apoptosis while neighboring cells remain unaffected [8]. The relative amount of expression of Fas on the surface of luteal cells might account for at least some of this selectivity and specificity, but this has not been directly evaulated in the CL. Instead, most studies to date have examined only gross expression of Fas mRNA or FasL in luteal tissue to propose a role for the Fas-FasL system in luteal function. In addition, potential mechanisms influencing Fas expression on the luteal cell surface have yet to be explored. Here we speculated cytoskeletal components, specifically intermediate filaments, regulate expression of Fas on the surface of luteal cells, and hence lend specificity to the process of Fas-induced apoptosis of luteal cells in the CL.

The cytoskeleton of cells consists of microtubules, microfilaments, and intermediate filaments. Intermediate filaments have a diameter ranging between 7–11 nm and consist of a family of five different subtypes [9]. One of the subtypes is the keratin-like proteins, which are found in epithelial tissues, including the steroidogenic cells of ovarian follicles and CL [10-16] . Keratin filaments are obligate heterodimers, forming filaments of an acidic keratin (type I, K9-K20), and a basic keratin (type II, K1-K8) [9,17]. The more prominent types of keratin filaments found in epithelial cells include filaments containing K7, K8, K18, and K19 [9]. In the bovine CL, K8/K18 filaments are observed in luteal cells throughout the estrous cycle, yet their relative expression diminishes with advancing age of the tissue [16]. Functionally, K8/K18 filaments provide structural integrity to cells, but they also influence intracellular transport mechanisms and signaling [18,19]. In particular, the expression of these filaments in certain types of epithelial cells provides a mechanism of resistance to apoptosis. For instance, K8/K18 filaments in hepatocytes impair cytokine receptor trafficking and cell surface expression [20-22]. Whether or not K8/K18 filaments similarly impair Fas expression on luteal cells has not been tested.

In the present study, the objective was to quantify Fas expression on bovine luteal cells during the early developmental (Day 5, postovulation) and late functional stages (Days 16–18, postovulation) of the CL, examine the relationship between luteal Fas and K8/18 filament expression, and assess the susceptibility of the luteal cells to cytokine-induced death. Our null hypothesis was that the surface expression of Fas on luteal cells does not differ between the two stages of corpora lutea (i.e., early vs. late stage CL). In addition, we tested whether the disruption of K8/K18 filaments in the luteal cells increases the cell surface expression of Fas, and thus their susceptibility to cytokine-induced apoptosis. Experimentally, cultures of bovine luteal cells from early and late stage CL were exposed acutely to acrylamide to disrupt the K8/K18 filaments. The effects of filament disruption on Fas expression and cytokine-induced apoptosis were then measured.

## Methods

### Collection of bovine corpora lutea for dissociation and Q-RTPCR

All animal studies described herein were approved by the UNH Institutional Animal Care and Use Committee (IACUC# 090205). Estrous cycles of Holstein dairy cows were monitored using transrectal ultrasonography, and corpora lutea (CL) were removed by colpotomy at days 5 (early stage; n=6 cows) and 16–18 (late stage; n=9 cows) postovulation (ovulation = day 0). Luteal cells obtained from CL at these two stages of luteal function express relatively high and low amounts of keratin intermediate filaments, respectively, based upon previous findings [16,23]. Prior to CL removal, blood samples were obtained by coccygeal venipuncture using heparinized tubes to measure plasma progesterone concentration and verify the relative stage of the estrous cycle. Corpora lutea and blood samples were transported to the laboratory on ice where the CL were extracted for total RNA (described below) and enzymatically dissociated using collagenase type I (Worthington, Lakewood, NJ) as described previously by others [24]. Following enzymatic dissociation, the viability of the luteal cells was estimated to be 88-93% as determined by trypan blue exclusion. The dissociated luteal cells were then either freshly-fixed in paraformaldehyde for flow cytometric analysis, or placed in serum-free culture for further experimentation (described below). The heparinized blood samples from the cows were centrifuged at 2056xg for 20 min at 4°C to obtain plasma, which was then frozen at −20°C until assayed for progesterone by radioimmunoassay (RIA) as described previously [25].

Total RNA was isolated from the two stages of bovine CL (n= 5–7 CL/stage) using a Quick-RNA^TM^ Mini Prep kit (Zymo Research, Irvine, CA). The total RNA was then purified from genomic DNA contamination using RQ1 RNase-Free DNase (Promega, Madison,WI). The purified total RNA was reverse-transcribed to synthesize cDNA using the qScript™ cDNA Synthesis Kit (Quanta Biosciences, Gaithersburg, MD). The cDNA was then used for subsequent quantitative real time polymerase chain reaction (Q-RTPCR) with SyBr Green detection (Quanta Biosciences, Gaithersburg, MD). Sequence-specific primers for bovine Fas and β-actin (an internal control gene), validated previously by Vickers et al.[26] and Taniguchi et al.[5], respectively, were as follows:

Forward and reverse primers, respectively for bovine Fas were: 5^′^-ATGGGCTAGAAGTGGAACAAAAC-3^′^ and 5^′^- TTCTTCCCATGACTTTGATACC-3^′^. Forward and reverse primers, respectively, for bovine β-actin were: 5^′^- GAGGATCTTCATGAGGTAGTCTGTCAGGTC-3^′^ 5^′^-CAACTGGGACGACATGGAGAAGATCTGGCA-3^′^.

A thermal cycler was used to conduct the Q-RTPCR with the cyclic conditions as follows: an initial Taq activation at 95°C for 2 min, followed by 40 cycles of 95°C for 1 second, 55°C for 30 seconds and 72°C for 30 seconds. All reactions were carried out on a 7500 Fast Real-Time PCR System. The data were collected during the last 30 seconds of cycling and the amplification signals of Fas transcripts were quantified using a standard curve based upon an absolute quantitation method. The results were expressed as a ratio of Fas relative to β-actin transcripts as the reference (i.e., internal control gene). Melting curve analysis was performed with conditions as follows: 95°C for 15 seconds, 60°C for 1 min, and 95°C for 15 seconds.

### Culture of bovine luteal cells and disruption of K8/K18 filaments with acrylamide

Freshly dissociated luteal cells were seeded in T25 flasks at a density of 2×10^6^ viable cells/flask and in 8-well microchamber slides at 2×10^4^ viable cells/well. The cells were cultured in serum-free Ham’s F12 culture medium (Invitrogen, Carlsbad, CA) supplemented with insulin, transferrin, selenium (ITS; 5μg/5μg/5ng/mL; Sigma Aldrich, St. Louis, MO) and gentamicin (20μg/mL; Invitrogen, Carlsbad, CA) and incubated at 37°C, 5% CO_2_ in air and 95% humidity overnight. The purity of the cultures under these serum-free conditions is estimated to be 70-75% steroidogenic cells because other types of cells (e.g., endothelial cells, fibroblasts, etc.) are unable to persist. The day after seeding, the flasks and chamber slides were rinsed and the conditioned medium replaced with fresh culture medium prior to treatments. Initial treatments consisted of flasks and chamber slides treated with either culture medium (control) or 5mM acrylamide (Fisher Scientific, Pittsburgh, PA) for 4 h to disrupt K8/K18 filaments and potentially increase the cell surface expression of Fas. Acrylamide is a selective, reversible, disrupter of K8/K18 filaments in mammalian cells [27] that under short-term culture conditions does not adversely affect microtubules [28,29], organelles (e.g., mitochondria, [30]), steroid synthesis [31], or cell viability [11]. After the initial 4 h treatment period, all flasks and chamber slides were rinsed twice and the medium replaced. Cells from several flasks were immediately prepared for flow cytometric analysis of Fas and K8/K18 expression as described below. The remaining flasks were treated with a cytokine cocktail containing bovine interferon-γ (IFN, 200 IU/mL; R&D Systems, Minneapolis, MN), murine tumor necrosis factor-α (TNF, 10ng/mL; US Biological, Swampscott, MA), and human recombinant soluble Fas ligand (FasL, 50ng/mL; R&D Systems, Minneapolis, MN) with a murine monoclonal anti-6x histidine cross-linking antibody (1mg/mL; R&D Systems, Minneapolis, MN) for 24 h to induce cell death. Others have previously shown this mixture of cytokines is appropriate, and necessary, to induce Fas-mediated death of bovine ovarian steroidogenic cells *in vitro*[5,6,26,32,33]. After 24 h incubation, the flasks were re-treated with the cytokine cocktail for an additional 24 h, prior to assessment of cytokine-induced cell death.

### Cell death counts

Cytokine-induced cell death in the cultured luteal cells was assessed at three different times during the experiment. The number of attached cells in five random microscopic fields of view was counted in all of the flasks prior to cytokine treatment using a 0.25 mm^2^ grid (initial cell counts). At 24 and 48 h after treatment, the number of attached cells in the flasks was again counted to estimate cell loss (post-treatment cell counts). All five fields of view per flask were averaged and the percent cell death was determined using the following equation:

(1) $$\begin{array}{ll} \%\;\text{Cell Death}= & (1-(\text{Post treatment cell counts}\\ & /\text{initial cell counts}))*100 \end{array}$$

### Culture of HepG2 cells

Murine hepatocytes were among the first cells used to demonstrate that disruption of K8/K18 filaments enhances Fas trafficking to the cell surface [20]. Here we utilized human hepatocyte carcinoma cells (HepG2 cells) to corroborate this finding under the experimental conditions used to disrupt K8/K18 filaments in bovine luteal cells with acrylamide. Briefly, HepG2 cells were seeded into T150 flasks at 2×10^6^ cells/flask. The cells were cultured in Eagle’s Minimal Essential Medium (Sigma Aldrich, St. Louis, MO) supplemented with 10% fetal bovine serum (JRH Biosciences, Lenexa, KS) and incubated at 37°C, 5% CO_2_ and 95% humidity. At approximately 70% confluency, the HepG2 cells were subcultured in T25 flasks using approximately 1x10^6^ cells/flask. The following day, the medium was changed and the cultures were exposed to vehicle (control) or 5mM acrylamide for 4 h. Following treatment, the cultures were prepared for flow cytometry to assess cell surface expression of Fas.

### Fixation of bovine luteal cells and HepG2 cells for flow cytometric analysis

Luteal cells from freshly dissociated CL and from serum-free culture were used to analyze Fas and K8/K18 filament expression by flow cytometry. For cells obtained through dissociation of CL, approximately 1.5x10^6^ cells/tube in 0.4mL of Ham’s F12 culture medium were centrifuged using screen-capped tubes (Ref # 352235, BD Falcon, San Jose, CA) for 5 min at 276xg, 4°C. The screened cells were then fixed for 2 h on ice by adding 0.4mL 2% paraformaldehyde to the cell suspension for a final concentration of 1% paraformaldehyde. After fixation, the cells either remained in fixative (for detection of cell surface Fas) or were rinsed twice with PBS and then permeabilized using 70% ethanol (for detection of total Fas and K8/K18 filament expression). Both the fixed and permeabilized cells were stored at 4°C and −20°C, respectively, until further processed for flow cytometry.

Luteal cells in serum-free culture and the HepG2 cells cultured in serum-containing conditions were fixed in a similar manner to the freshly isolated luteal cells described above. Briefly, the flasks of cells were rinsed twice (5 min each) with Hank’s Balanced Salt Solution (Sigma Aldrich, St. Louis, MO), followed by two quick washes with trypsin-EDTA (Cell Gro Mediatech, Manassas, VA). After the second trypsin-EDTA rinse, the remaining trypsin was removed and the flasks were left for 10 min. The trypsinized cells were then collected in Ham’s F12 culture medium containing 10% fetal bovine serum (JRH Biosciences, Lenexa, KS), centrifuged for 5 min at 276xg, 4°C and resuspended in Ham’s F12 culture medium without serum. As above, approximately 1.5x10^6^ cells/tube were centrifuged using screen-capped tubes for 5 min at 276xg, 4°C. The filtered cells were fixed for 2 h on ice in 1% paraformaldehyde and either remained in fixative (detection of cell surface Fas; luteal and HepG2 cells) or were permeabilized with 70% ethanol (detection of total Fas and K8/K18 filaments; luteal cells only). Both the fixed and the permeabilized cells were stored at 4°C and −20°C, respectively, until analyzed by flow cytometry.

### Flow cytometric analysis of Fas and K8/K18 expression

Fixed cells (i.e., luteal and HepG2) were washed twice (5 min each) with phosphate buffered saline with 0.1% bovine serum albumin (PBS-BSA) and centrifuged at 276xg for 5 min at 4°C between each wash. Following the second wash, the cells were stained for Fas using a mouse anti-human Fas antibody (clone CH11; Millipore, Billerica, MA; diluted 1:25 with PBS with 10% normal goat serum) or an identical concentration of nonspecific, IgG1 isotype (clone MOPC-21; Sigma) as a control. The cells were incubated in primary antibody overnight at 4°C and then washed twice (5 min each) with PBS-BSA with spins at 276xg for 5 min at 4°C between each wash. Detection of the primary antibody was achieved fluorescently using a goat anti-mouse Alexa 488-conjugated IgG secondary antibody (Invitrogen, Carlsbad, CA) diluted 1:200 with PBS-BSA with 10% normal goat serum. For detection of K8/K18, luteal cells from CL dissociation and from culture were washed twice (5 min each) with PBS-BSA and spun at 276xg for 5 min at 4°C between each wash. The cells were then incubated for 1 h at 37°C with a mouse anti-human K18 FITC-conjugated antibody (clone CY-90; Sigma Aldrich, St. Louis, MO; diluted 1:100 with PBS- BSA). Previously we have shown K18 dimerizes with K8 such that targeting of K18 is sufficient for the detection of K8/K18 filaments in bovine luteal cells [16]. Quantification of cells expressing Fas and K8/K18 was accomplished using a 4 color, dual laser FACScalibur flow cytometer (Becton Dickinson Biosciences, San Jose, CA) with a 488nm argon laser for FITC/Alexa 488 excitation. The negative controls, either IgG1-FITC (for K18 detection) or Alexa-488 secondary antibody only (for Fas detection), were used to set the fluorescence gating to 1% positive controls prior to analysis. The cells were recorded on the FL-1 filter at no more than 800 events/second with a total of 10,000 recorded events. Data were collected using Cell Quest (Becton Dickinson Biosciences, San Jose, CA) and graphs of the results were generated using WinMDI 2.9 software (Scripps Institute, La Jolla, CA). Mean fluorescence intensity (MFI), a measure of staining intensity for each cell, was calculated using the following equation:

(2) $$\begin{array}{ll} \text{MFI}= & (\text{Geometric mean of sample}\\ & –\text{Geometric mean of negative control})\\ & /\text{Geometric mean of negative control} \end{array}$$

### Microscopic evaluation of K8/K18 filaments and microtubules in bovine luteal cells

Bovine luteal cells cultured in microchamber slides were used to evaluate microscopically the efficacy and specificity of acrylamide as a disrupter of K8/K18 filaments. The cells were rinsed twice with PBS, fixed using 4% paraformaldehyde in PBS for 20 min on ice, and then stored in PBS at 4°C until permeabilized with methanol and analyzed for K8/K18 expression and microtubule expression (negative control) by fluorescent microscopy. Briefly, the previously-fixed luteal cells were rinsed twice with PBS-BSA followed by a 1 h block/permeabilization step with 0.3% triton x-100 in PBS containing 10% normal goat serum (Vector Labs, Burlingame, CA) and 3% BSA. The slides were rinsed 3 × 5 min with PBS-BSA and incubated overnight at 4°C with either a mouse anti-human K18 monoclonal antibody (clone CY-90; Sigma Aldrich, St. Louis, MO; diluted 1:800 in PBS-BSA with 10% normal goat serum), or a mouse anti-bovine alpha-tubulin monoclonal antibody (clone 236–10501; Invitrogen, Carlsbad, CA; diluted 1:200 in PBS-BSA with 10% normal goat serum). The following day, after 3 × 5 min washes with PBS-BSA, fluorescent detection of the K18-containing filaments or tubulin-containing microtubules was achieved by incubating the slides with a goat anti-mouse Alexa 488-conjugated IgG antibody (K18; Invitrogen, Carlsbad, CA) or a goat anti-mouse Texas Red-conjugated antibody (microtubules; Santa Cruz, Santa Cruz, CA). Both secondary antibodies were diluted 1:200 in PBS-BSA with 10% normal goat serum (Vector Labs, Burlingame, CA). The slides were counterstained with 4',6-diamidino-2-phenylindole (DAPI) mounting medium (Vector, Burlingame, CA) and then coverslipped.

### Statistical analysis

The data were analyzed by 1-way or 2-way ANOVA followed by Tukey’s multiple comparison test using the general linear model of Systat 12.0 (Point Richmond, CA). Results are expressed as mean ± SEM, with each experiment repeated three to nine times (i.e., n= 3–9). For experiments requiring cultured cells, the cells were cultured in triplicate for a given experiment and were derived from individual CL or from a frozen stock of cells (HepG2 cells). Thus, the total number of experiments (n=) is equivalent to the total number of CL or frozen aliquots (HepG2) used to establish the cultures; shown in figure legends). Differences among means at a value of P<0.05 were considered statistically significant.

## Results

### Fas expression is greater for bovine luteal cells of early stage CL compared to late stage CL

**Figure 1:**
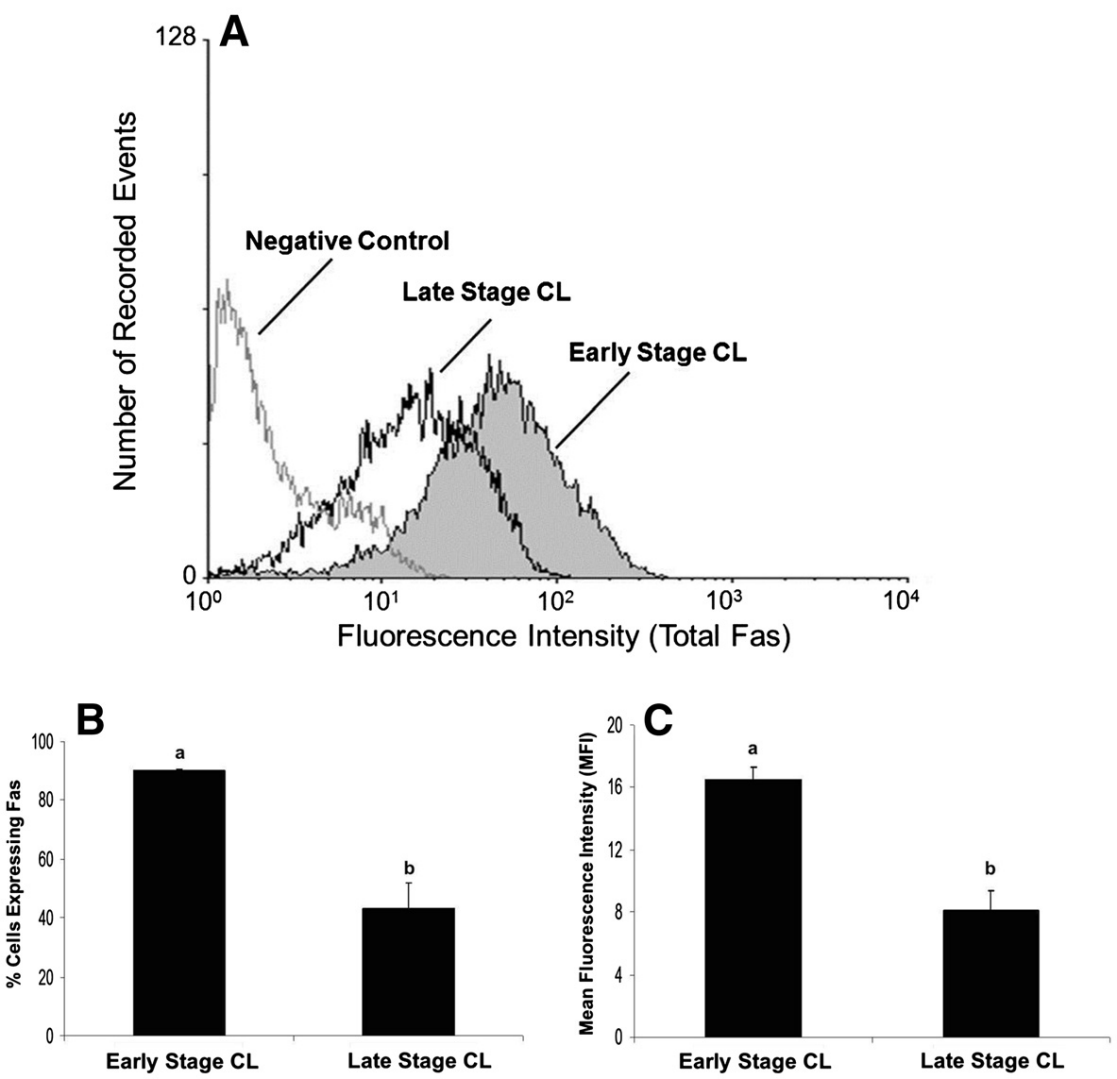
**Flow cytometric analysis of total Fas in cells of early and late stage bovine CL.** A representative histogram depicting the total amount of Fas detected in bovine luteal cells of early and late stage CL is shown (**Figure 1A**). Relative number of cells expressing total Fas is depicted for early versus late stage CL (**Figure 1B**). Relative mean fluorescence intensity (MFI) is also depicted for the two stages of CL (**Figure 1C**). Values shown are mean ± SEM; different letters indicate significant differences (P<0.05; n=6-9 CL/stage).

**Figure 2:**
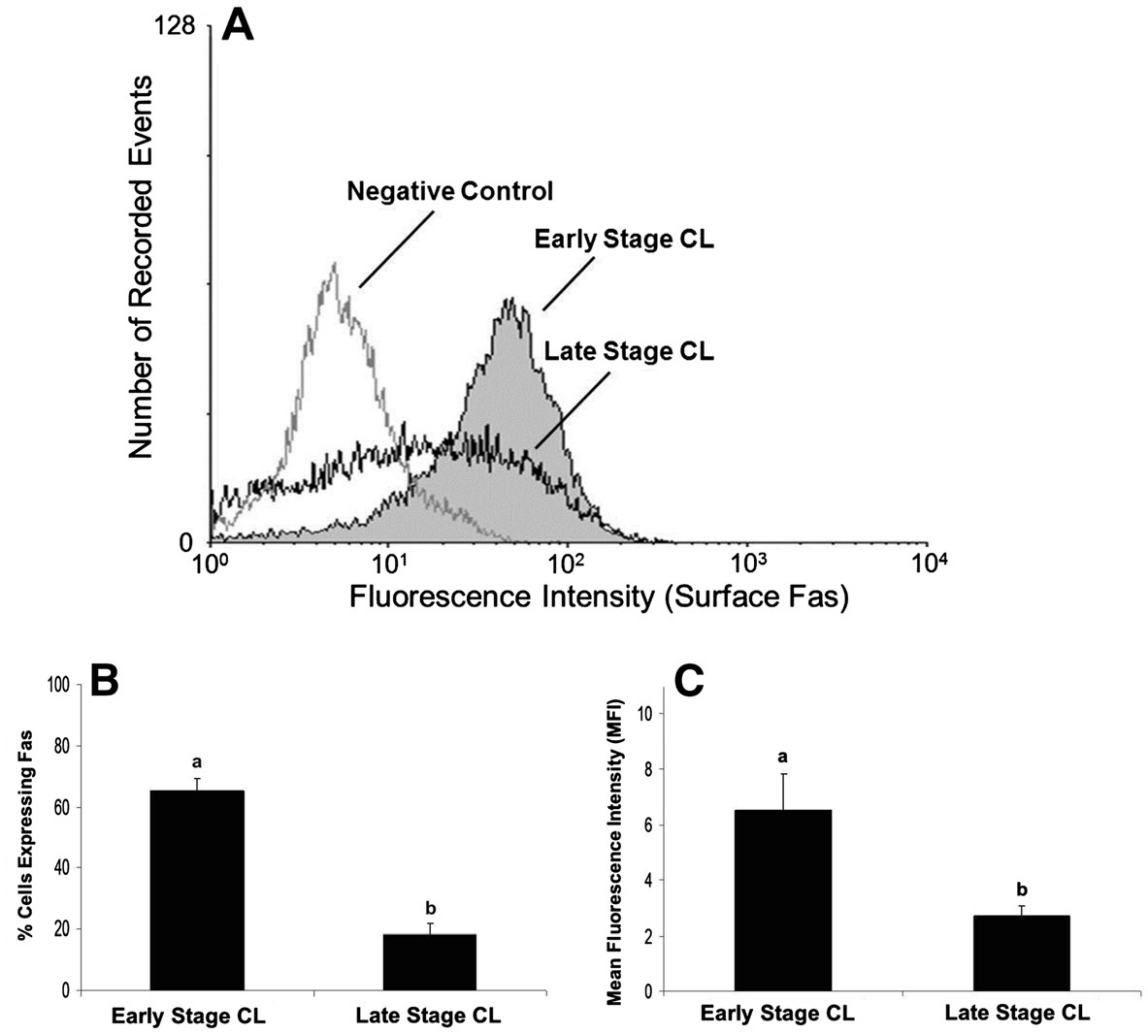
**Flow cytometric analysis of Fas expression on the surface of cells of early and late stage bovine CL.** A representative histogram depicting the expression of Fas on the surface of bovine luteal cells of early stage and late stage CL is shown (**Figure 2A**). Relative number of cells expressing Fas on the cell surface is depicted for early versus late stage CL (**Figure 2B**). Relative mean fluorescence intensity (MFI) for the two stages of CL is also shown (**Figure 2C**). Values shown are mean ± SEM; different letters indicate significant differences (P<0.05; n=6-9 CL/stage).

**Figure 3:**
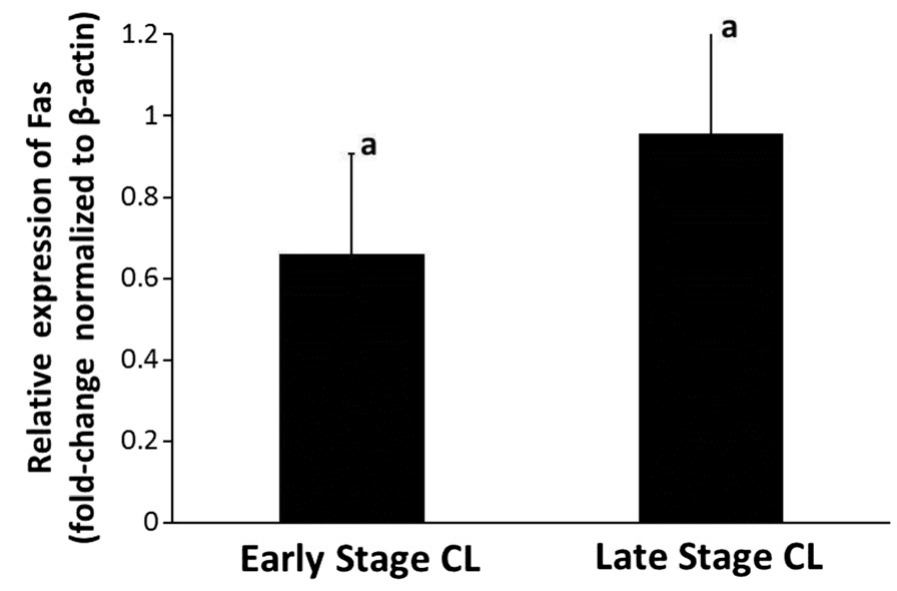
**Relative expression of Fas mRNA in bovine CL during the early and late stages of the estrous cycle.** Values shown are mean ± SEM fold-change of Fas expression (normalized using β-actin). Different letters indicate significant differences (P<0.05; n=5-7 CL/stage).

Freshly dissociated luteal cells from early and late stage CL were characterized for total Fas expression (Figure 1) and expression on the cell surface (Figure 2) relative to a non-specific IgG control. Measurement of plasma progesterone revealed the cows used to obtain early stage CL had lower systemic progesterone than cows used for late stage CL (1.8 ± 0.2 versus 5.9 ± 0.7 ng/ml, respectively; P<0.05, n=6-9 CL/stage). However, a higher percentage of luteal cells expressed total Fas in early stage CL compared to late stage CL (Figure 1A, P<0.05). Mean fluorescence intensity (MFI), a measure of staining intensity for each cell, was also higher among cells from early stage CL compared to late stage CL (Figure 1C, P<0.05). Similarly, the expression of Fas on the cell surface was greater for cells of early stage CL compared to late stage CL (Figure 2B, P<0.05), as was MFI (Figure 2C, P<0.05). Overall, quantification of the percentage of cells expressing Fas on the cell surface relative to total Fas expression revealed cells from early stage CL express the majority of Fas on the cell surface (76%), whereas less than half these cells from late stage CL do so (47%). In terms of relative steady-state concentrations of Fas mRNA in the luteal tissue, Q-RTPCR indicated there was no difference between early versus late stage CL (Figure 3; P>0.05, n=5-7 CL/stage).

Interestingly, a comparison of Fas expression for freshly dissociated luteal cells versus luteal cells placed in culture for 24 h revealed that culture alone substantially increased the relative cell surface expression of Fas for cells of both early and late stage CL. Cell surface expression of Fas increased from ~65% to ~97% as a result of culture for cells of early stage CL (P<0.05, n=4 expts.), and from ~18% to ~66% for cells of late stage CL (P<0.05, n=4 expts.).

### K8/K18 filament expression is increased in bovine luteal cells of early stage CL compared to late stage CL

**Figure 4:**
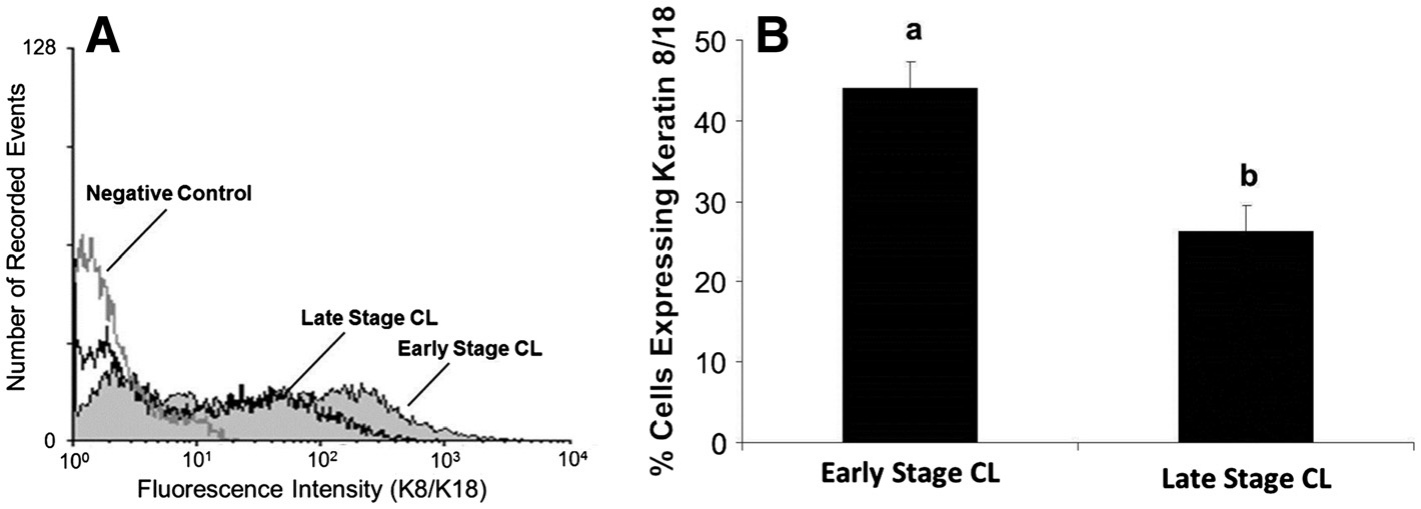
**Flow cytometric analysis of K8/K18 expression in cells of early and late stage bovine CL.** A representative histogram depicting the expression of K8/K18 filaments in bovine luteal cells of early stage and late stage CL is shown (**Figure 4A**). The relative number of cells expressing K8/K18 filaments is depicted for early stage CL versus late stage CL (**Figure 4B**). Values shown are mean ± SEM; different letters indicate significant differences (P<0.05; n=6-9 CL/stage).

A higher percentage of freshly dissociated luteal cells from early stage CL expressed K8/K18 filaments than late stage CL (Figure 4, P<0.05). Average number of cells expressing K8/K18 filaments in early stage CL was 46% compared to 26% for late stage CL (Figure 4B). In contrast to what was observed for cell surface expression of Fas, culture of luteal cells for 24 h did not enhance K8/K18 expression in cells of early or late stage CL. Relative percentage of K8/K18-positive cells was 46% vs. 49% for freshly dissociated vs. cultured cells, respectively, in early stage CL, and was 26% vs. 23% for freshly dissociated vs. cultured cells, respectively, in late stage CL (P>0.05, n=4 expts., data not shown).

### Acrylamide-induced disruption of K8/K18 filaments does not enhance cell surface expression of Fas or cytokine-induced apoptosis

**Figure 5:**
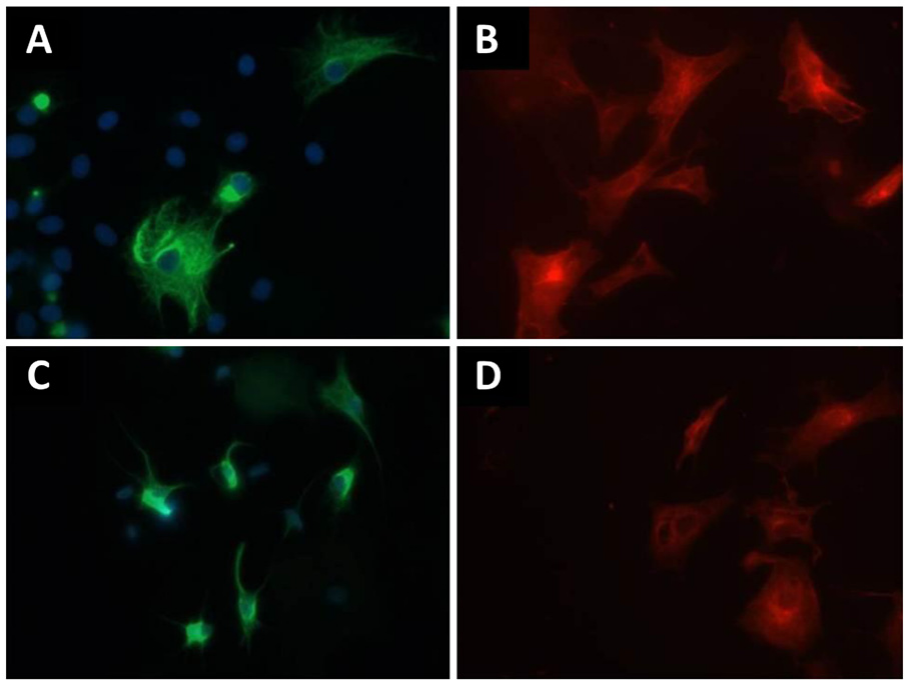
**Fluorescent detection of K8/K18 filaments and microtubules in control and acrylamide-treated cultures of bovine luteal cells.** K8/K18 filaments (green fluorescence) and microtubules (red fluorescence) were immunostained in cultured bovine luteal cells following 4 h exposure to vehicle (Control, **Figure 5A** and **B**) or 5mM acrylamide (**Figure 5C** and **D**). Cells in control cultures exhibited a filamentous, K8/K18 intermediate filament network which spanned the cytoplasm (**Figure 5A**). Microtubles of these cells was similarly filamentous (red fluorescence; **Figure 5B**). Conversely, cells of acrylamide-treated cultures exhibited peri-nuclear aggregation of K8/K18 filaments (**Figure 5C**), yet the microtubules were unaffected (**Figure 5D**). Magnification: 40x.

Exposure of cultured bovine luteal cells to acrylamide disrupted K8/K18 filaments without adversely affecting microtubule organization (Figure 5). Cells in control cultures exhibited extensive, filamentous networks of K8/K18 staining (Figure 5A) that became aggregated around the perinuclear region of the cells following acrylamide exposure (Figure 5C). Conversely, microtubule organization when compared between control and acrylamide-treated cultures remained unaffected (Figure 5B and D, respectively). In addition, there was no observable effect of stage of CL on these outcomes, and the acrylamide treatment overall had no effect on the number of cells expressing K8/K18 filaments, luteal cell viability or progesterone secretion (P>0.05; n=2-4 CL/stage, data not shown).

**Figure 6:**
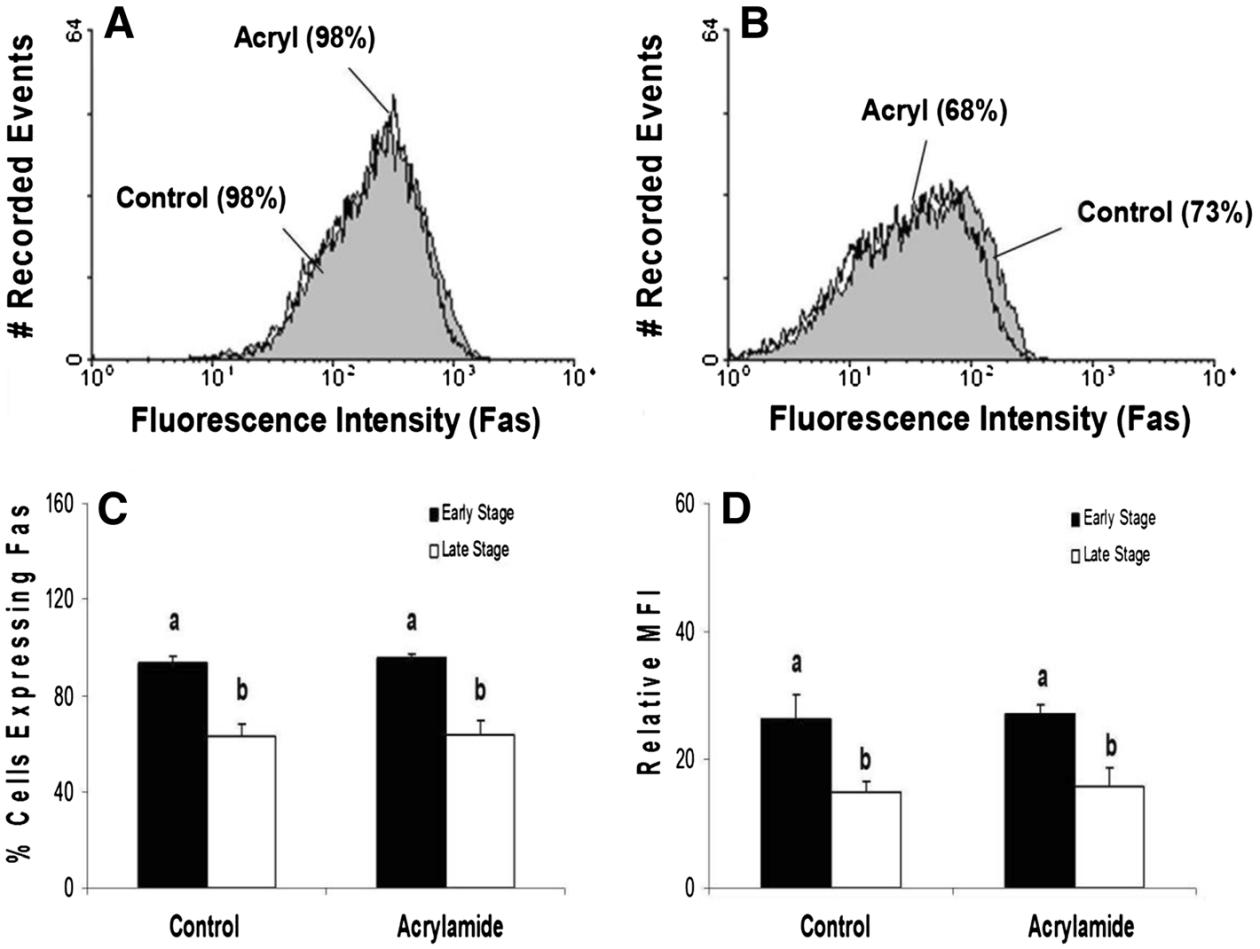
**Flow cytometric analysis of Fas expression on the surface of cells of early and late stage bovine CL following K8/K18 filament disruption with acrylamide.** Representative histograms depicting the expression of Fas on the surface of bovine luteal cells of early stage and late stage CL are shown (**Figure 6A** and **B**, respectively). The relative percentage of cells expressing Fas on the cell surface is depicted for early versus late stage CL, and for control versus acrylamide-treated cells (**Figure 6C**). Relative mean fluorescence intensity (MFI) is also depicted for the two stages of CL and the treatment conditions (**Figure 6D**). Values shown are mean ± SEM; different letters indicate significant differences (P<0.05; n=4 CL/stage).

**Figure 7:**
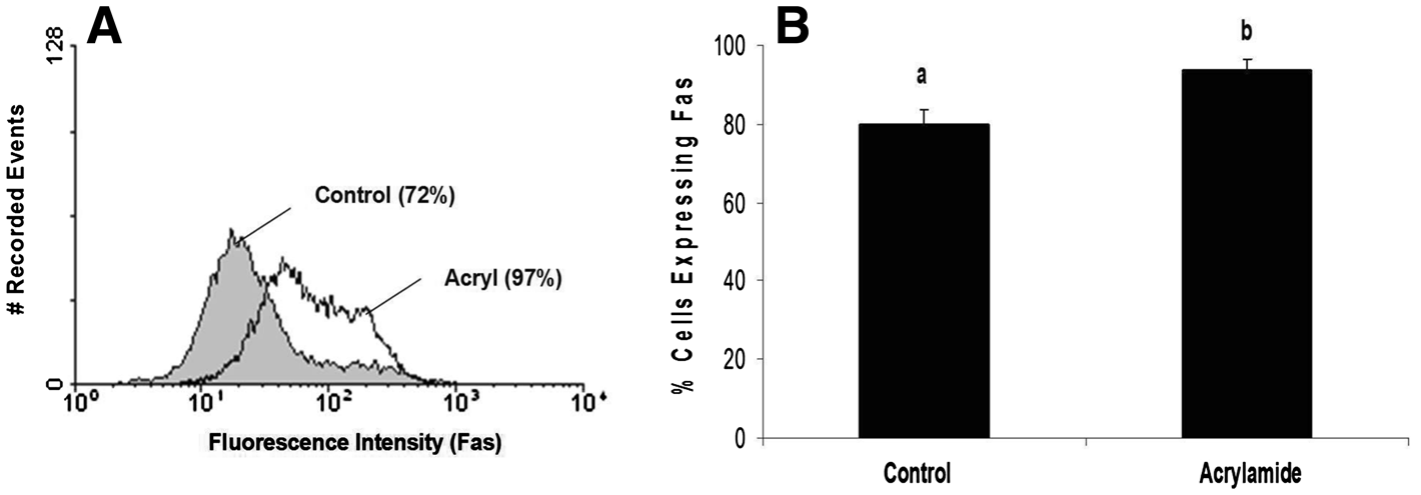
**Flow cytometric analysis of Fas expression on the surface of HepG2 cells following K8/K18 filament disruption with acrylamide.** A representative histogram depicting the expression of Fas on the surface of HepG2 cells is shown (**Figure 7A**). The relative percentage of cells expressing Fas on the cell surface is depicted for control versus acrylamide-treated cells (**Figure 7B**). Values shown are mean ± SEM; different letters indicate significant differences (P<0.05; n=3 expts.).

Although acrylamide disrupted K8/K18 filaments, no increase in the cell surface expression of Fas was observed for luteal cells of either stage of CL (Figure 6A-C; P>0.05). Moreover, K8/K18 filament disruption failed to enhance Fas cell surface expression on specific cells, as reflected by the lack of change in relative MFI (Figure 6D; P>0.05). Consistent with the observations of freshly isolated luteal cells, cultured luteal cells of early stage CL expressed higher amounts of Fas on the surface than cultured cells of late stage CL (Figure 6C and D; P<0.05). In contrast, disruption of K8/K18 filaments in HepG2 cells, using identical experimental conditions to those for bovine luteal cells, increased the number of cells expressing Fas on the cell surface (Figure 7; P<0.05).

**Figure 8:**
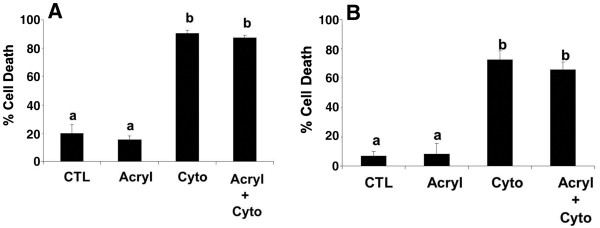
**Cell death in cultured luteal cells from early and late stage bovine CL following exposure to acrylamide and cytokines.** The relative percentage of cells undergoing death after 48 h exposure to cytokines (Cyto) is depicted for early and late stage CL (**Figure 8A** and **B**, respectively). The cultures were also exposed to a 4 h pretreatment with 5mM acrylamide (Acryl) to disrupt K8/K18 filaments before cytokine treatment. Values shown are mean ± SEM; different letters indicate significant differences (P<0.05; n=3 expts.).

Exposure of the cultured bovine luteal cells for 48 h to a cytokine cocktail consisting of IFN, TNF, and FasL induced cell death, as expected, but there was no effect of K8/K18 disruption by acrylamide (P>0.05) or stage of CL (P>0.05, n= 4 CL/stage) on this outcome (Figure 8). Similar results were observed when the luteal cells were exposed to cytokines and acrylamide for only 24 h (data not shown).

## Discussion

The current study is the first to directly measure relative changes in the expression of Fas on the surface of bovine luteal cells across the estrous cycle. The observation of quantifiably higher Fas receptor expression on luteal cells from early stage compared to late stage CL was unexpected and somewhat contrary to what was anticipated based upon earlier published studies. In these studies, the investigators examined the gross expression of Fas mRNA [5,7] and protein [34,35] in ovarian tissues, without reference to cell-specificity and they found that Fas increased only in CL undergoing regression. In the current study, Fas protein was quantified for individual cells obtained from CL following tissue dissociation and cell culture, and then analyzed using flow cytometry. Similarly, Fas mRNA expression for the two stages of CL was measured by Q-RTPCR. The current methods are arguably more quantitative than the mRNA detection, immunoblot analysis, and immunohistochemistry methods described in the cited studies, but fall short of identifying specific cell type(s) known to exist within the CL. Nevertheless, dissociation of the luteal tissue and establishing serum-free culture conditions, as described, removes many of the various cell types, while enriching the population of luteal steroidogenic cells. Thus, we suggest the pattern of Fas expression observed in the current study is essentially representative of the luteal steroidogenic cell population within the bovine CL at the two extremes of the estrous cycle. Moreover, our observation of no measureable difference in relative steady-state amounts of mRNA for Fas in early versus late stage CL, as evaluated by Q-RTPCR, is consistent with a previously published study [5].

Overall, a 72% decline in the number of bovine luteal cells expressing Fas at their cell surface, and a 59% decline in the density of Fas expressed at the cell surface across the estrous cycle was observed. Total Fas expression (surface and intracellular) for freshly isolated cells was higher for early stage CL than late stage CL. A similar difference in Fas surface expression was observed for cultured luteal cells, but was further enhanced by culture alone. Exposure of the cultured cells to the cytokine cocktail of IFN + TNF + FasL, however, resulted in similar estimates of cell death for both stages of CL. This indicates cultured luteal cells from both stages of CL are equally vulnerable to cytokine-mediated cell death despite clear differences in the cell surface expression of Fas.

The observation that Fas expression is elevated on luteal cells of early stage CL without further enhancing their susceptibility to cytokine-induced death indicates mechanisms exist to protect the cells against Fas-induced apoptosis. For instance, a soluble secreted isoform of Fas has been identified in other tissues that sequesters FasL prior to binding at the target cell surface, thus preventing cell death [36-38]. This isoform of Fas lacks the transmembrane domain of wild-type Fas, causing it to be secreted rather than expressed on the surface of cells [38]. The murine ovary expresses a soluble form of Fas, which has protective effects [36]. Thus, it is possible a soluble form of Fas exists within the bovine CL to modulate the effect of elevated Fas expression in early stage CL as seen in the current study. Certainly this possibility merits additional exploration.

Another intrinsic “protective” mechanism of cells of early stage bovine CL might include the expression of membrane-bound splice variants of the Fas receptor. The cytokine TRAIL (TNF-related apoptosis-inducing ligand) for example, which is structurally similar to FasL, binds to receptors, DR4 and DR5, yet membrane-bound decoy receptors also exist for TRAIL. These receptors, named DcR1 and DcR2, have a cytoplasmic domain structurally similar to DR4 and DR5, respectively, but lack the intracellular death domain necessary for transmitting an apoptotic signal [39-41]. Recently, Sugimoto and coworkers identified a putative Fas decoy receptor, DcR3, in granulosa cells of porcine ovaries [42]. Similar to DcR1 and DcR2, DcR3 contains an extracellular and cytoplasmic domain similar to wild-type Fas, but lacks the intracellular death domain. Unlike soluble Fas, the decoy receptor is expressed on the plasma membrane and retains its ability to bind FasL, but does not induce cell death [43]. It is tempting to speculate that a decoy receptor of Fas may exist on bovine luteal cells, explaining the high prevalence of Fas expression observed for cells of early stage CL, but not late stage CL, in the current study. Further research is needed to determine whether or not a Fas decoy receptor exists within the bovine ovary, and to explore its possible role in ovarian function.

Alternatively, enhanced expression of Fas on cells of early stage CL can be explained by a non-apoptotic or even proliferative role of Fas in the early stage CL. In recent years, diverse non-apoptotic functions of Fas have been documented [44], such as the acceleration of liver regeneration after partial hepatectomy [45], the induction of cell migration and invasiveness of apoptotic-resistant tumor cells [46], and the stimulation of cardiomyocyte hypertrophy [47]. The ability of Fas to control the fate of the cell likely hinges on the regulation of Fas-induced downstream signaling events, such as activation/inhibition of the ERK, JNK, p38, and NF-κB pathways. These same pathways have suggested roles in luteal cell function and fate [5,48-50], but their influence on the developing early CL, especially in the context of elevated Fas expression, is unknown. Overall, the concept that Fas might facilitate development of the CL is consistent with the premise suggested by Pate and Keyes [51], in which immune-response mechanisms exist within the ovary to abate damaging inflammatory responses caused by dead or dying cells. In the current scenario, these cells would arise from postovulation trauma during the initial development of the CL.

In the present study, acrylamide selectively disrupted the K8/K18 filaments in the luteal cells, but did not enhance Fas expression or otherwise influence Fas-mediated cell death. In effect, this result did not support the concept that K8/K18 filaments influence Fas trafficking at the cell surface. However, acrylamide causes intermediate filaments to only partially disassemble and undergo acute dephosphorylation [30]. Dephosphorylation provokes a 50% loss of phosphate from the keratin protein which corresponds with the morphological changes observed for intermediate filament expression [27]. At best, the dephosphorylation event is transient, and the striking changes in intermediate filament organization are reversible. In fact, the filaments re-establish their ‘net-like’ organization generally within 12 h after acrylamide removal [29], and complete rephosphorylation occurs within 18 h [27]. In the current investigation, the K8/K18 filaments of bovine luteal cells were exposed to acrylamide for only 4 h. This was sufficient time to noticeably disrupt the filaments, but perhaps insufficient to sustain a change in Fas trafficking or in downstream signaling that would otherwise enhance cell death. It is noteworthy, however, that these same conditions increased Fas expression on HepG2 cells in the current study. For the time-being, we cannot reject the possibility that K8/K18 filaments influence events downstream from Fas binding; however, it seems unlikely that the filaments directly impair Fas expression on the cell surface as has been suggested in other studies [20-22].

## Conclusions

In conclusion, the elevated expression of Fas on cells of early stage bovine CL compared to late stage bovine CL raises a provocative question concerning the physiological role(s) of Fas in the corpus luteum. Although there is little doubt about the apoptotic function of this receptor during luteal regression, its purpose during early luteal development has yet to be defined. We suggest, as others do, that a broader view of Fas-mediated activities merits consideration, including the need to identify the signaling components linking Fas to non-apoptotic pathways. These insights may provide new targets to influence fertility, and treat diseases such as inflammation and cancer.

## Abbreviations

CL: Corpus luteum or corpora lutea; FasL: Fas ligand; K: Keratin; mRNA: messenger RNA; mM: millimolar; PBS-BSA: Phosphate buffered saline containing bovine serum albumin; Q-RTPCR: Quantitative real-time polymerase chain reaction.

## Competing interests

The authors declare that they have no competing interests.

## Authors’ contributions

AD and JF equally carried out the cell culture studies, performed the flow cytometric analyses, conducted the microscopy work and assisted in the preparation of the manuscript. AB conducted the Q-RTPCR analysis. DT conceived of the study, participated in its design and coordination, and drafted the manuscript. All authors read and approved the final manuscript.
